# Sustainable Micro-Scale Extraction of Bioactive Phenolic Compounds from *Vitis vinifera* Leaves with Ionic Liquid-Based Surfactants

**DOI:** 10.3390/molecules25133072

**Published:** 2020-07-06

**Authors:** Giulia Mastellone, Idaira Pacheco-Fernández, Patrizia Rubiolo, Verónica Pino, Cecilia Cagliero

**Affiliations:** 1 Dipartimento di Scienza e Tecnologia del Farmaco, Università degli Studi di Torino, I-10125 Torino, Italy; giulia.mastellone@edu.unito.it (G.M.); patrizia.rubiolo@unito.it (P.R.); 2 Laboratorio de Materiales para Análisis Químicos (MAT4LL), Departamento de Química, Unidad Departamental de Química Analítica, Universidad de La Laguna (ULL), 38206 Tenerife, Spain; ipacheco@ull.edu.es (I.P.-F.); veropino@ull.edu.es (V.P.); 3 Instituto Universitario de Enfermedades Tropicales y Salud Pública de Canarias, Universidad de La Laguna (ULL), 38206 Tenerife, Spain

**Keywords:** bioactive phenolics, grapevine, green extraction, sample preparation, microwave-assisted extraction, ionic liquid

## Abstract

This paper proposes a new sustainable and simple strategy for the micro-scale extraction of phenolic compounds from grapevine leaves with analytical purpose. The method is based on a microwave-assisted solid-liquid extraction approach (MA-SLE), using an aqueous solution of an ionic liquid (IL)-based surfactant as extraction phase. The method does not require organic solvents, nor any clean-up step, apart from filtration prior to the injection in the analytical system. Two IL-based surfactants were evaluated, and the method was optimized by using experimental designs, resulting in the use of small amounts of sample (100 mg) and extraction phase (2.25 mL), low concentrations of the selected 1-hexadecyl-3-butyl imidazolium bromide IL (0.1 mM), and 30 min of extraction time. The proposed methodology was applied for the determination of the polyphenolic pattern of six different varieties of *Vitis vinifera* leaves from the Canary Islands, using high-performance liquid chromatography and photodiode array detection for the quantification of the compounds. The proposed MA-SLE approach was greener, simpler, and more effective than other methods, while the results from the analysis of the leaves samples demonstrate that these by-products can be exploited as a source of natural compounds for many applications.

## 1. Introduction

Environmental sustainability is nowadays an object of global attention, and indeed regulatory institutions are developing strategies and designing road maps to promote the efficient use of resources, the recovery of biodiversity, and the reduction of waste and pollution, encouraging a Circular Economy approach [1]. In this respect, the valorization of agrifood by-products can play a key role. Agrifood by-products such as seeds, peels, roots, leaves, and stems, which are produced during cultivation and food processing, generate large volumes of waste and represent a disposal problem for the agriculture and food industries. At the same time, the unexploited plant materials are often rich in bioactive phytochemical compounds, such as terpenoids, phenolic compounds, and alkaloids, with high bioactivity against several common diseases. These phytochemicals can be exploited as green and renewable sources of nutrients and bioactive compounds for the feed, functional food, and food supplement industries [2,3,4,5,6].

In this context, wine production and viticulture represent one of the main and most widespread agro-economic activities, but, at the same time, they produce huge amounts of residues. Many grapevine by-products, such as grape pomace, seeds, and stems, have been investigated by both the phytochemical and bioactivity points of view [4,7,8]. Besides these vine parts, vine leaves are another important residue generated in high amounts (from 1 to 7.5 t/ha) by vineyard pruning [9]. Grapevine leaves are traditionally used as food [10], animal feed [11], ingredients of dietary supplements [12], and in cosmetics [7]. As well as the other parts of the plant, they are chiefly characterized by phenolic compounds. Phenolic acids and flavonols are the main classes of compounds, but small amounts of stilbenes (such as resveratrol), flavan-3-ols, and anthocyanins (mainly in red autumn leaves) can also be present [8,10,13,14]. The beneficial properties of grapevine leaves are attributed to these phenolic compounds and are mainly correlated to their well-known antioxidant activity [8,12,14]. In particular, Acquadro et al. [15] showed in 2020 that, thanks to their characteristic phenolic pattern, grapevine leaves and green pruning residues represent a potential source of natural compounds with valuable antioxidant properties. To sum up, they could be used as natural antioxidants and functional food ingredients, reducing, at the same time, the environmental impact of viticulture. Therefore, it is of utmost importance to determine the phenolic profile of these by-products before their exploitation.

Environmental sustainability has also reached the laboratories, and thus Green Analytical Chemistry (GAC) emerged with the aim of improving the analytical methodologies. According to GAC principles, the minimization of energy, reagents and sample consumption and the elimination of hazardous reagents, automation, and direct analysis are the key points to develop alternative and environmentally friendly analytical methods [16]. Despite these purposes, it is fair to recognize that direct analysis is not possible in most cases [17]. In particular, sample preparation steps are needed for the isolation of compounds from challenging solid samples, such as plants, characterized by the presence of numerous compounds and interferences that are difficult to remove. In general, plant extracts are obtained by solid-liquid extraction approaches (SLE) that also require other pretreatment steps (drying and grinding) and post treatment procedures, such as filtration, concentration, or purification. These requirements lead to numerous steps and the consumption of large amounts of organic solvents, being as a result a tedious and environmentally unfriendly procedure [18].

In this sense, green extraction methods have been proposed over the years for plant analysis, including miniaturized approaches, which comprise the use of low amounts of both sample and extraction phase [17,19]. Regarding the time and energy consumption, the main strategy has been the incorporation of heating and stirring methods, such as microwaves and ultrasounds, to speed up the procedure, while ensuring increases in the extraction efficiency [20]. The development of solvent-free approaches or the use of alternative solvents, to reduce the consumption of solvents coming from petroleum sources, is one of the most important research lines nowadays [18]. Among the explored options, ionic liquids (ILs) arise as adequate candidates thanks to their unique set of physicochemical properties [21]. These molten salts, formed by the combination of organic cations and organic or inorganic anions, present negligible vapor pressure at room temperature and, contrary to conventional organic solvents, do not produce volatile organic compounds. Given their impressive synthetic tunability, IL-based surfactants can be prepared by incorporating long aliphatic chains into their structures. Aqueous solutions of these IL derivatives have attracted much attention in extraction approaches due to their solvation properties derived from their ability to undergo micellization at concentrations much lower than conventional surfactants with similar structures (in turn, highly minimizing the amounts required) [22]. Although ILs and IL-based surfactants have been included in methods for the extraction of bioactive compounds from plants, those approaches are still characterized for the use of organic solvents and additional cleaning steps, thus reducing the entire greenness of the procedure [23,24,25].

Taking into account the above considerations, the aims of this study were, on one side, the development of a truly green, fast, and reliable method for the micro-scale extraction of bioactive phenolic compounds from grapevine leaves for analytical purpose, and on the other side, the evaluation of the phytochemical pattern of the leaves of a set of varieties of *Vitis vinifera* L. (harvested in the Canary Islands), in view of their possible exploitation as a source of antioxidant compounds. The proposed method consisted of a focused-microwave-assisted solid-liquid extraction approach (MA-SLE), using micellar aqueous solutions of an IL-based surfactant, followed by HPLC and photodiode array detection (PDA). Different ILs were evaluated, while the experimental conditions were optimized by using an experimental design to obtain the maximum extraction efficiency. Furthermore, the proposed method was compared in terms of extraction capacity and greenness with an ultrasound-assisted (UA-SLE) approach and other methods reported in the literature for the grapevine leaves analysis.

## 2. Results and Discussion

### 2.1. Preliminary Extraction Screening Using Different IL-Based Surfactants

One of the most important variables to consider when developing the MA-SLE method is the nature of the IL-based surfactant to extract the target compounds. Supplementary Materials Figure S1 shows the chemical structure for the IL-based surfactants evaluated in this study, which were selected considering their critical micelle concentration (CMC) values and their performance under cytotoxicity studies. Furthermore, both ILs were compatible with HPLC analysis due to their solubility in the mobile phase, thus not requiring a back-extraction of the IL extract before the injection in the instrument. The C_10_Gu-*Cl* IL-based surfactant is characterized by its low cytotoxicity in comparison with conventional surfactants and more common IL-based surfactants containing imidazolium moieties, and this has been demonstrated by cell viability assays [26,27]. The length of the apolar tail of this IL confers surface active properties with CMC values ranging from 18.6 [27] to 22 mM [28], depending on the technique used for its determination. In the case of C_16_C_4_Im-*Br* IL-based surfactant, the long alkyl chain in its structure (Supplementary Materials Figure S1B) leads to a really low CMC [26], with a value of 0.1 mM [29].

In order to evaluate the extraction capacity and behavior of the IL-based surfactants for this specific application, both ILs were used in the MA-SLE method, using the Italian cultivar mix, and following the experimental conditions previously reported for a similar MA-SLE procedure with food samples, but with slight modifications [30]. With comparison purposes, the UA-SLE method that has been previously applied for this batch of Italian cultivars was also performed by following the experimental conditions described in Section 3.4.4.

**Figure 1:**
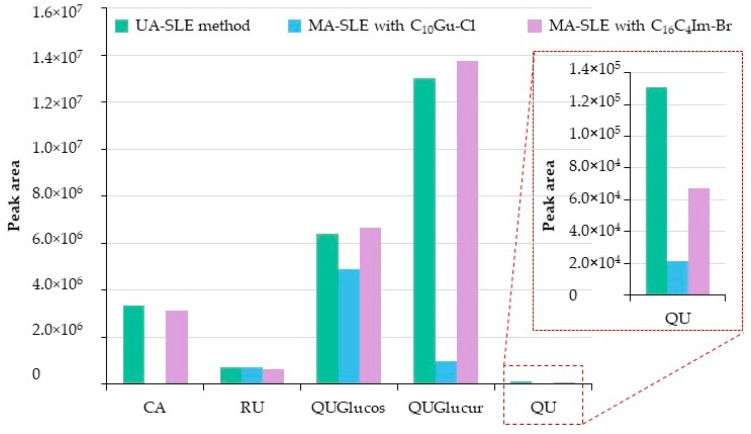
Preliminary screening of the extraction performance (evaluated as peak area) of the proposed MA-SLE method, using C_10_Gu-*Cl* and C_16_C_4_Im-*Br* IL-based surfactants in comparison with the UA-SLE method used as reference method. The preliminary experimental conditions for the MA-SLE method were 100 mg of leaves, 2.25 mL of IL-based surfactant aqueous solution at 2.5 mM for C16C4Im-*Br* and 300 mM for C_10_Gu-*Cl*, MW treatment at 70 °C, and 50 W for 30 min. Legend: CA, caftaric acid; QU, quercetin; QUGlucos, quercetin-3-*O*-glucoside; QUGlucur, querceting-3-*O*-glucuronide; RU, rutin.

Figure 1 includes the peak areas obtained for the target phenolic compounds, using the conventional UA-SLE method and the MA-SLE with both IL-based surfactants. It can be observed that the results obtained with the C_16_C_4_Im-*Br* IL-based surfactant and MA-SLE method are similar to those obtained with the UA-SLE method for all the compounds, except for QU, for which the conventional method provided a higher peak area. It is important to point out that the conditions used for the UA-SLE method are those previously optimized for this specific application [15], while the MA-SLE method was performed under random preliminary conditions (which can be clearly improved if optimized). Furthermore, QU is present in a very low amount, and a lower extraction will not significantly influence the bioactivity of the extracts. Regarding C_10_Gu-*Cl* IL-based surfactant, it revealed a limited ability of extraction for the majority of compounds in comparison with the imidazolium IL and the UA-SLE method, in particular for the CA, while similar results were observed for QUGlucur.

Therefore, taking into account the results of this screening study, C_16_C_4_Im-*Br* IL-based surfactant was selected for the development of the MA-SLE method. Although C_10_Gu-*Cl* presents lower cytotoxicity in comparison with C_16_C_4_Im-*Br* (but that of C_16_C_4_Im-*Br* still acceptable if compared with conventional surfactants [26]), the CMC of the imidazolium IL-based surfactant is significantly lower (0.1 mM). This characteristic allows for the use of small amounts of the IL-based surfactant to exploit its surface-active properties. This may benefit the extraction procedure and goes in accordance with GAC guidelines, due to the reduced amounts of reagents involved in the procedure.

### 2.2. Optimization of the MA-SLE Method Using the C_16_C_4_Im-*Br* IL

**Figure 2:**
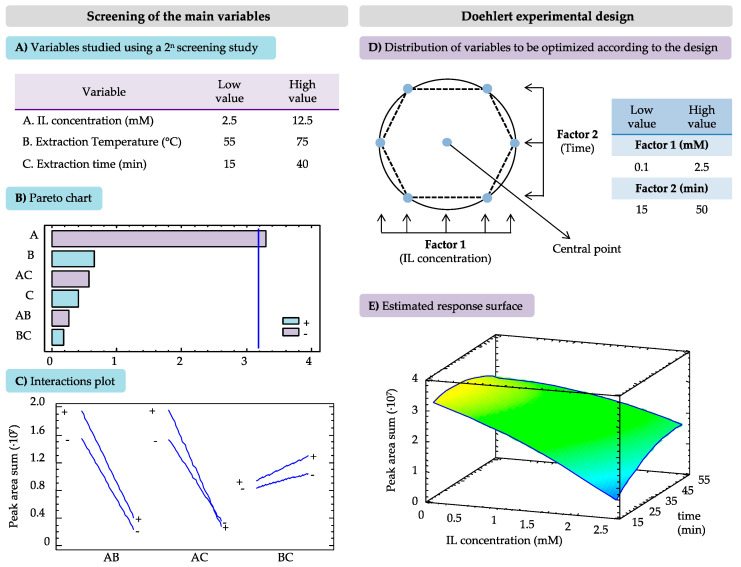
Results obtained in the screening of the main variables and in the Doehlert experimental design, during the optimization of the MA-SLE method. (**A**) Variables considered in the screening process. (**B**) Effects of the main factors in the resulting peak area sum for all the compounds. (**C**) Interaction among factors for all the compounds. (**D**) Spatial distribution of the experimental points in the Doehlert design. (**E**) Estimated response surface for peak areas sum for all the compounds, considering IL concentration and time of extraction factors.

The optimization of the MA-SLE method by using aqueous solutions of the C_16_C_4_Im-*Br* IL-based surfactant as extraction medium was performed with an experimental design, to reduce the number of experiments and to determine the interactions between the variables that affect the extraction efficiency toward the target analytes. Several experimental parameters in the method were fixed with the aim of avoiding time- and energy-consuming steps, guaranteeing the use of low amounts of sample and reagents in the procedure, while considering other operational limitations. Therefore, 0.100 g of leaves sample and 2.25 mL of the IL aqueous solution were used, while the centrifugation step to separate the sample from the extraction solution was set to 5 min at 2504× *g*. Taking into account previous studies dealing with MA-SLE methods, the MW power was fixed at 50 W, since higher power values do not lead to better extraction efficiencies [30]. Thus, the variables considered for the optimization of the process were the IL-based surfactant concentration (mM), and the extraction temperature (°C) and time (min) during the MW treatment. Figure 2 reports the results obtained in the screening of the main variables and in the Doehlert experimental design, during the optimization of the MA-SLE method.

A screening test was initially carried out to determine which of those parameters significantly affect the extraction, with the purpose of reducing the number of variables to optimize in the experimental design. A full two-level factorial design (2n, with *n* = 3 variables) was used for this preliminary study, which comprises eight experiments combining the minimum and maximum values considered for each factor. Moreover, three replicates of the central point (intermediate value for each variable) were carried out, in order to monitor the reproducibility of the method. The IL-based surfactant concentration was evaluated in a range high above the CMC of the IL (CMC = 0.1 mM), and therefore the low and high limits were set at 2.5 and 12.5 mM, respectively. Regarding the extraction temperature, a minimum value of 55 °C was required for the MW treatment, while the maximum value was set in accordance with the literature data that show the possible degradation of phenolic compounds above 75 °C [31]. The limits for the extraction time were selected for being common times reported in the literature, in MA methods. Figure 2A shows the ranges in which the three variables were studied, while Supplementary Materials Table S1 includes the design matrix for the screening study. Once the 11 experiments were performed, the extraction efficiency was evaluated in terms of the sum of the peak areas obtained for all the target compounds at their corresponding wavelength.

The Pareto chart included in Figure 2B shows that the IL-based surfactant concentration is the most influential variable on the method, and, interestingly, it is inversely proportional to the peak area value. This means that lower concentrations of C_16_C_4_Im-*Br* (always above the CMC) allowed us to extract higher amounts of the analytes. This behavior may be associated with the increased viscosity of the extraction solution that has been observed when high concentrations of long alkyl chain ILs are used, which may limit the transfer of the analytes from the sample to the extraction phase [32,33]. Both the extraction temperature and time during the MW treatment had a positive effect on the peaks area, but to a lesser extent than the IL-based surfactant concentration. However, in the interactions plot shown in Figure 2C, it is observed the intersection between extraction time and IL concentration, demonstrating the interaction between these two factors when they are varied within the range studied. Based on these results, the extraction temperature was set at 70 °C, while the IL concentration and the extraction time were the factors considered for further optimization by using a Doehlert experimental design.

**Figure 3:**
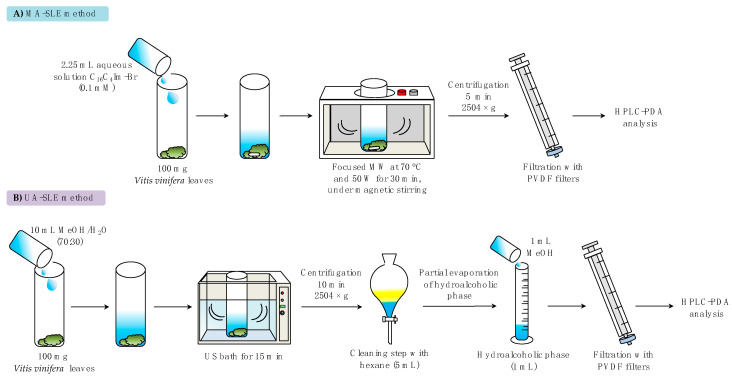
Scheme of the extraction procedures from *Vitis vinifera* leaves: (**A**) proposed MA-SLE method and (**B**) UA-SLE method used with comparison purposes, which was previously reported for this specific application [15].

Figure 2D shows a scheme of the spatial distribution of the different experimental points, according to the Doehlert design, for two variables selected in this study. Supplementary Materials Table S2 includes the matrix of the experiments carried out, which consist of nine experiments considering the hexagonal geometry of the design, as well as three replicates of the central point. This sophisticated experimental design requires fewer experiments, because the experiments are more efficient and uniformly distributed within the experimental domain, in comparison with other response surface designs commonly used in optimization studies [34]. As it was demonstrated in the screening study, the IL-based surfactant concentration had the strongest influence, and it was studied at five levels in a lower concentration range, from 0.1 (its CMC value) to 2.5 mM. The extraction time was assessed at three levels in the same range, from 15 to 50 min. The response surface included in Figure 2E was obtained by considering the sum of the peak area for all the compounds in each experiment and fitting the values to the polynomial equation, using the Lagrange’s criterion [34]. It can be observed that the maximum enhancement of the signal is obtained when the IL-based surfactant concentration was the lowest (0.1 mM), with the extraction time between 25 and 35 min. Taking into account these results, the optimum conditions for the MA-SLE method for the extraction of the target phenolic compounds were the following: 0.100 g of leaves sample, 2.25 mL of an aqueous solution containing the C_16_C_4_Im-*Br* IL-based surfactant at 0.1 mM, extraction temperature of 70 °C at 50 W, and extraction time of 30 min. Figure 3 reports the comparison between the schemes of the extraction procedures with the proposed MA-SLE method (Figure 3A) and the reference UA-SLE method (Figure 3B).

#### Influence of Chlorophyll Content on the Method Performance

One of the most important issues regarding the analysis of plant samples is the content of strongly binding molecules, such as pigments, which eventually could compromise the analytical performance of the chromatographic column [35]. For this reason, these pigments, mainly chlorophylls, are usually removed before the analysis in the HPLC by using solvents or an additional cleanup step based on solid-phase extraction methods. Indeed, in the UA-SLE method previously reported for the same application developed in this study, a liquid-liquid extraction (LLE) step using an apolar solvent is accomplished right after the UA extraction for the elimination of chlorophylls, as shown in Figure 3B [15]. Petroleum-based solvents, which are commonly used for the removal of these interfering substances, present many drawbacks, such as high volatility, flammability, and toxicity, with an important impact on both the environment and human health [36]. Moreover, these toxic solvents are not entirely effective for the extraction of these pigments, and part of them still remains in the aqueous or alcoholic phase. Therefore, the incorporation of this tedious additional step not only increases the analysis time but also compromises the sustainability of the entire process. In this sense, one of the goals of the present study was the elimination of this step, to ensure the development of a greener method in terms of both energy consumption and elimination of toxic solvents.

The presence of chlorophylls is easy to evaluate, since they provide a bright green color to the final extracts. In the case of the C_10_Gu-*Cl* IL-based surfactant used in the screening study, the extract after the MA-SLE method (under random preliminary conditions) exhibited a strong green color, revealing the presence of the pigments. Interestingly, the extract obtained with the optimum MA-SLE method using the C_16_C_4_Im-*Br* IL-based surfactant (which uses low concentrations of the IL) presented a similar yellowish color to that of the UA-SLE method after the cleaning LLE step, thus supporting the scarce presence of chlorophylls in the extract. It is important to highlight that the extraction was carried out in an almost pure aqueous medium, with a small amount of IL (0.1 mM). This medium is not favorable for the extraction of non-polar interferences, besides chlorophylls, such as lipids or other non-polar compounds, which may lead to the presence of important interferences further affecting the quantification results.

### 2.3. Quantification of Phenolic Compounds by HPLC-PDA

After the optimization of the method and correct identification of the target compounds in the chromatogram, their quantification was required in order to determine the concentration of the analytes in the different samples. The external standard calibration method was used for the quantification of the analytes in the samples.

Supplementary Materials Table S3 includes several quality analytical figures of merit of the method, including the linearity range, the calibration sensitivity (evaluated as the calibration slope), determination coefficients (R^2^), limits of detection (LOD), and limits of quantification (LOQs), for each compound. The LODs were experimentally determined by decreasing the concentration of the analytes until a signal-to-noise ratio (S/N) of 3 was obtained. The LOQs were estimated as 10/3 times the LODs and experimentally verified by injecting the standards at the predicted concentrations. Thus, the LODs ranged from 0.5 mg·L^−1^ for QU to 3.0 mg·L^−1^ for CA, while the LOQs were between 1.7 and 5.0 mg·L^−1^ for the group of analytes. Considering the LOQ values obtained, the calibration curves were obtained by using seven calibration levels, in the range of 5 to 500 mg·L^−1^, for all the phenolic compounds, except for CA, for which the calibration range reaches 650 mg·L^−1^. The R^2^ values were higher than 0.992 in all cases, which demonstrates the good linearity obtained in the studied calibration range. Regarding the sensitivity, QU exhibited the highest calibration slope, which agrees with the low LOD obtained compared with the rest of the analytes.

#### 2.3.1. Comparison between UA-SLE and MA-SLE Method Using Italian Cultivars

Once the calibration curves were obtained, the analysis of the samples was carried out by injecting the extracts after applying the proposed MA-SLE or the UA-SLE method to the Italian leaf samples. Each extraction was performed in triplicate, to express the results as the mean concentration together with the standard deviation (SD), which is used as a measure for the precision of the method. Furthermore, given the high concentration of some analytes in the samples, their quantification required the dilution of the extracts with ultrapure water (1:20) to obtain signals included within the calibration range.

Table 1 includes the concentrations of the target phenolic compounds found in the Piedmont leaves (Italian cultivar) after their analysis by the UA-SLE-HPLC-PDA and the proposed MA-SLE-HPLC-PDA method [37,38,39]. The results were expressed as mean values ± SD in mg·g^−1^. A similar absolute composition was obtained by using both methods, in which the most abundant compound in the Piedmont variety was QUGlucur, followed by CA and QUGlucos. Indeed, these results agree with those obtained in the previous study in the literature that determined the phenolic content of the same sample [15]. However, it is important to highlight that the concentrations obtained after the analysis by the MA-SLE method are higher compared with those obtained with the UA-SLE approach, especially for QUGlucur and QUGlucos. For these two phenolic compounds, the difference is more significant, obtaining concentrations of 32.1 ± 0.4 mg·g^−1^ and 10.3 ± 0.4 mg·g^−1^ with the MA-SLE method, and values of 17 ± 2 mg·g^−1^ and 5 ± 1 mg·g^−1^ by using the UA-SLE approach, respectively. These values demonstrate that the proposed MA-SLE method with the IL-based surfactant provides better extraction efficiency. Supplementary Materials Figure S2 shows the chromatograms obtained after the injection of the extracts obtained from the UA- and MA-SLE methods in the HPLC-PDA system. As it can be observed, the profiles of the chromatograms are similar with both methods, while the peaks corresponding to CA, QUGlucur, and QUGlucos are higher in the MA-SLE extract.

Furthermore, as shown in the quantification results, lower SD values were obtained with the MA-SLE procedure. The precision of the proposed method was also evaluated with the relative standard deviation values (%RSD, *n* = 3) by performing three extractions of the Italian cultivars in the same day (intra-day) and in three non-consecutive days within a week (inter-day). The intra-day RSD values ranged between 4.6 and 11%, while the inter-day RSD values varied from 6.8 to 15% for QUGlucos and RU, respectively.

Apart from the differences in the extraction performance of both methods, it is important to highlight other advantages of the proposed MA-SLE method over the already reported UA-SLE for the same application. As shown in Figure 3, the proposed MA-SLE method comprises fewer steps, making the process less tedious and faster due to the elimination of the cleanup stage, during which the chlorophylls are removed and the solvent is partially evaporated. Moreover, the proposed procedure is more sustainable, since the extraction phase consists of an aqueous solution of an IL-based surfactant at a quite low concentration level (less than 10 mg of IL is required in each extraction). Besides, the absence of organic solvents in the entire procedure, in comparison with other common MA-SLE methods reported in the literature for plant analysis [23,25,40,41,42,43,44,45,46], makes it comply with one of the most important requirements set by the GAC. Although other MA-SLE methods using ILs have been previously proposed in the literature for the extraction of bioactive compounds from plant material, those methods usually incorporate a cleanup step prior the injection in the analytical system [24,47,48], or they require the dilution of the extract with organic solvents [49,50,51,52]. Moreover, most of these applications do not fit with the miniaturization recommendations set by GAC [17], with the amount of plant sample and IL solution around 0.5–1 g and 10 mL, respectively [24,49,50,51].

#### 2.3.2. Analysis of Canarian Leaves Varieties by MA-SLE-HPLC-PDA

Once the adequate performance of the extraction method was demonstrated, it was used for the determination of the phenolic composition profile of different varieties of *Vitis vinifera* leaves. Some reports in the literature indicate a variation in the phenolic composition of the by-products of *Vitis vinifera*, depending on the variety, the characteristics of the soil, water availability, cultural practices, and climatic conditions, including temperature and luminosity [53,54,55]. Therefore, six different Canarian varieties were selected as samples (see Supplementary Materials Table S4). Three of them produce red wine (Listán Negro (LN), Negra Moll (NM), and Tintilla (T)), while the other three yield white wine (Listán Blanco (LB), Malvasía Lanzarote (ML), and Moscatel Alejandría (MA)). Moreover, with the aim of investigating the influence of geographic position of the cultivars, the phenolic composition profile obtained was also compared with the Italian mix cultivar previously analyzed in this study.

Each pool of leaves was analyzed with the proposed MA-SLE-HPLC-PDA method, in triplicate, to express the concentration results with their corresponding SD. The data obtained from these analyses are reported in Table 1 and plotted in Supplementary Materials Figure S3.

The MA sample had the highest phenolic content among all the varieties, while LN presented the lowest amount of total target phenolic compounds. The most significant differences were observed for the content of QUGlucur, since an average value around 33 mg·g^−1^ was obtained, but the T and MA varieties exhibited concentrations of 62 ± 9 mg·g^−1^ and 65 ± 9 mg·g^−1^, respectively. In the case of RU, the highest content was found in ML variety, with a concentration of 4.8 ± 0.2 mg·g^−1^. The concentration of QUGlucos was around 8.5 mg·g^−1^, except for the lower content found in LN and T varieties, with values of 3.4 ± 0.4 mg·g^-1^ and 5.5 ± 0.2 mg·g^−1^, respectively. The amount of the remaining phenolic compounds CA and QU was similar in all the samples, with QU being the least abundant among all the analytes considered. In general, all the varieties exhibited a similar phenolic pattern as shown in Supplementary Materials Figure S3.

The extracts of several of the analyzed Canarian cultivars presented additional peaks apart from those of the target phenolic compounds. In particular, a peak at 14.8 min was found in the NM variety with a similar peak area as RU. Considering the UV-Vis spectrum of that peak and the data of the analysis of *Vitis vinifera* leaves in the literature [15], this new peak can be tentatively identified as Vitilagin or Isovitilagin. A peak at 10.7 min before CA was also observed with a high peak area in the LN variety, but also in the profiles of ML and NM. Two more peaks were detected in all the samples close to QUGlucur, with retention times of 23.0 and 29.0 min. The identification and quantification of these unknown peaks were not possible, due to the lack of a mass spectrometer detector. In any case, the peak areas of these compounds were also considered, together with those of the target analytes, for the statistical elaboration of the results, with the aim of assessing if there is any correlation between the phenolic content and the variety of *Vitis vinifera* leaves analyzed. Supplementary Materials Figure S4 includes the results obtained from the Principal Component Analysis (PCA) and Hierarchical Cluster Analysis (HCA) performed by using the peak area data. In the PCA analysis, the first component explained 46% of the variance of the dataset, while the second component explained 27%. In Supplementary Materials Figure S4A, a clear discrimination between two groups was observed, considering the first component: LB, MA, and T varieties and ML, LN, and NM cultivars. As shown in the loading plot included in Supplementary Materials Figure S4B, this discrimination is due to the high amounts QUGlucur found in the first group of cultivars, together with the lower amount of QUGlucos in comparison with the varieties composing the other group (ML, LN, and NM). Furthermore, a slight discrimination can be observed in the second component between the varieties yielding white wines (ML, LB, and MA) and red wines (NM, LN, and T), which may be mainly related to the lower signal of Vitilagin/Isovitilagin obtained for the white wine varieties and the slightly higher amount of CA found in the red wine types. Despite the low number of data considered, it is interesting to point out that the heatmap obtained from the HCA using one minus Pearson’s correlation coefficient (Supplementary Materials Figure S4C) showed the same distribution of the data and confirmed the correlation between the phenolic content of the two groups described in the first component of the PCA.

The results obtained from the analysis of the Canarian leaves were compared with the phenolic composition profile of the Italian leaves mix used for the development of the proposed MA-SLE method. As it is shown in Supplementary Materials Figure S3, a similar distribution of the target compounds was observed, in which QUGlucur was the most abundant compound, followed by CA and QUGlucos. The content of RU was low in all the samples, while QU represented a very small part of the total phenolic composition. The specific levels found for each compound in the Italian cultivars, together with the concentrations reported in the literature for other varieties of *Vitis vinifera* leaves from Serbia [39], Italy [38], and Brazil [37], are included in Table 1. The QU concentration values obtained for the Canarian samples and the Italian mix analyzed in the present study are similar to those reported for other varieties, with values around 0.20 mg·g^−1^. However, it is important to highlight that all the Canarian leaves presented a notable higher content of RU in comparison with the other varieties, for which concentrations lower than 1 mg·g^−1^ were obtained, while amounts up to 4.8 mg·g^−1^ were found in the samples from the Canary Islands. It is also interesting to point out that, although the total phenolic content of the Brazilian samples is considerably lower, the most abundant compound in these leaves is QUGlucur, as it was observed for the samples analyzed in the present study. Recently, it was also demonstrated that all the compounds considered in the phenolic content profile present a similar antioxidant effect [15]. Therefore, the antioxidant activity of the product is mainly related to the total concentration of phenolic compounds in the sample. Given these results, it is possible to assume that the geographic distribution slightly influences the composition of the phenolic profile, and the Canarian varieties that presented the highest phenolic content may have some significant outcomes with respect to the valorization of these by-products.

## 3. Materials and Methods 

### 3.1. Chemicals and Reagents

The synthesis of the IL-based surfactants required 1*H*-pyrazole-1-carboxamidine-hydrochloride (99%), decylamine (≥99%), 1-butylimidazole (98%), and 1-bromohexadecane (97%), which were supplied by Sigma-Aldrich (Steinheim, Germany). Ethanol LiChrosolv^®^ grade LC, methanol CHROMASOLV™, and hexane SupraSolv^®^ were purchased from Merck KGaA (Darmstadt, Germany), while ethyl acetate (extra pure) and isopropanol (extra pure) were both supplied by Fisher Scientific (Fair Lawn, NJ, USA). Acetonitrile CHROMASOLV™ from Honeywell Riedel-de Haën™ (Seelze, Germany), ultrapure water with a resistivity of 18.2 MΩ·cm, which was obtained from a Milli-Q water purification system (Bedford, MA, USA), and glacial acetic acid (99%, Sigma-Aldrich, Steinheim, Germany) were used to prepare the chromatographic mobile phase.

Five phenolic compounds acquired from Phytolab (Vestenbergsgreuth, Germany) were determined in this study: caftaric acid (CA, ≥97%), quercetin (QU, ≥95%), quercetin-3-*O*-glucoside (QUGlucos, ≥90%), querceting-3-*O*-glucuronide (QUGlucur, ≥90%), and rutin (RU, ≥90%). Supplementary Materials Table S5 includes the chemical structures and physicochemical properties of the analytes. Individual standard solutions were prepared by dissolving them in methanol at 500 mg·L^−1^, except for CA, which was prepared at 650 mg·L^−1^. Working standard solutions containing all the analytes were prepared at different concentrations in acetonitrile, to obtain the calibrations curves. All of these solutions were kept protected from light and refrigerated at 4 °C.

### 3.2. Instrumentation and Equipment

A Sartorius analytical balance (Madrid, Spain) with a minimum readability of 0.1 mg was used to weight reagents, standards, and samples. A hot-plate magnetic stirrer, an RV 10 digital V rotary evaporator from IKA-Werke GmbH & Co. (Staufen, Germany), a Telstar^®^ Lyophilizer model (Barcelona, Spain), a focused CEM Discover microwave oven (CEM Corporation, Matthews, NC, USA), an ultrasound bath model UCI-150 from Raypa (Barcelona, Spain), and a centrifuge model 5702 from Eppendorf (Hamburg, Germany) were used to carry out the extraction procedures.

Pyrex^®^ (Staffordshire, UK) centrifuge tubes of 15 mL (9.5 cm × O.D. 2 cm), magnetic stir bars of 12.7 mm × 3.2 mm from Sigma-Aldrich, a Fortuna Optima^®^ glass syringe of 2 mL (Sigma-Aldrich, Steinheim, Germany), and PVDF (polyvinylidene fluoride) syringe filters (13 mm, 0.2 µm) from Whatman (GE Healthcare, Buckinghamshire, UK) were also used in the extraction method.

The separation and identification of the target analytes were performed in an HPLC system consisting of a Varian ProStar 230 solvent delivery (Palo Alto, CA, USA) and a Varian Prostar 330 photodiode array detector (PDA). The chromatographic system was also equipped with a manual injection system, with a Rheodyne 7725i valve and a 5 μL loop supplied by Supelco (Bellefonte, PA, USA). The separation was accomplished by using an Ascentis^®^ RP-Amide (15 cm × 4.6 mm × 5 µm) column, which was protected with a guard column with the same characteristics, both supplied by Supelco. A 25 μL syringe from Hamilton (Reno, Nevada, USA) was used for the manual injection in the HPLC-PDA system.

Experimental designs and their elaboration, and principal component analysis, were carried out by using Statgraphics^®^ Centurion XV.I software, while the heatmap was created by using Morpheus software (https://software.broadinstitute.org/morpheus). Excel software (Microsoft Office, v.2016) was employed for the remaining calculations.

### 3.3. Plant Material

Different types of *Vitis vinifera* leaves were employed in this study, depending on the experiments. All the samples were freeze-dried, grounded in a mortar, to obtain a fine powder, and stored in a dryer, to prevent degradation.

The optimization of the proposed MA-SLE method was accomplished by using a mixture of Italian cultivars from Piedmont (Italy) [15].

The quantification studies were performed by using six Canarian cultivars, which were kindly provided by the Canary Agronomic Research Institute (ICIA, Tenerife, Spain). The different cultivars included 3 white wine varieties: Malvasía Lanzarote (ML), Moscatel Alejandría (MA), and Listán Blanco (LB); and 3 were red wine varieties: Tintilla (T), Negra Moll (NM), and Listán Negro (LN). Supplementary Materials Table S4 includes the characteristics of these samples, which were grown in the same plot of land and collected when all the leaves were in the same physiological state (April 2019).

### 3.4. Procedures

#### 3.4.1. Synthesis of IL-Based Surfactants

Decylguanidinium chloride (C_10_Gu-*Cl*) was synthetized by following a previously reported procedure [27]. Briefly, 1.70 mL of decylamine and 1.24 g of 1*H*-pyrazole-1-carboxamidine hydrochloride were dissolved in 2.5 mL of ethanol. This solution was left under magnetic stirring at 35 °C for 48 h. Then, the IL was washed 3 times with 2.5 mL of ethanol, which was evaporated under vacuum (50 °C 150 mbar) until the viscous IL was obtained.

The IL-based surfactant 1-hexadecyl-3-butyl imidazolium bromide (C_16_C_4_Im-*Br*) was synthetized as was previously described in the literature [29]. Briefly, 12.4 g of 1-butylimidazole and 33.6 g of 1-bromohexadecane were refluxed in 20 mL of isopropanol at 70 °C for 24 h under stirring. The solvent was removed under vacuum (60 °C, 140 mbar), and the product was dissolved in 25 mL of water. It was washed 5 times with 15 mL of ethyl acetate, and, finally, water was evaporated under vacuum (80 °C, 70 mbar), and the product was dried in a vacuum oven, to obtain the solid IL-based surfactant.

#### 3.4.2. HPLC-PDA Analysis

The analysis of the extracts and determination of the target phenolic compounds was performed with an HPLC-PDA system, using acetonitrile and ultrapure water containing 0.1% (*v*/*v*) of acetic acid as mobile phases. The best separation was obtained with an RP-Amide (15 cm × 4.6 mm × 5 µm) column, using an initial composition of 5% (*v*/*v*) of acetonitrile, which was increased to 100% (*v*/*v*) in 45 min, at a flow rate of 1 mL·min^−1^. The detection and quantification wavelengths were 360 nm for all the analytes, except for CA, for which the selected optimum was 320 nm. The identification of the phenolic compounds in the chromatograms of the extracts was carried out while considering their retention times and their UV spectra and comparing them with those of the respective standards. The identification was further confirmed by comparing the obtained profiles with those of a previous study in which these compounds were determined in the same grapevine leaves samples by using both HPLC-PDA and LC coupled with tandem mass spectrometry (MS/MS) [15].

#### 3.4.3. MA-SLE Method Using an IL-Based Surfactant

In the optimum MA-SLE method developed in this study, 0.100 g of leaves sample was placed in a centrifuge tube, together with the stir bar, and 2.25 mL of 0.1 mM of an aqueous solution of the IL-based surfactant C_16_C_4_Im-*Br* IL was added. The mixture was introduced in the focused microwave system and programmed at 70 °C and 50 W for 30 min, while applying magnetic stirring. Once the extraction was completed, the mixture was left to cool to room temperature and centrifuged for 5 min at 2504 × g. The supernatant was collected by using a Pasteur Pipette and filtrated (using PVDF syringe filters), to be injected in the HPLC-PDA system.

#### 3.4.4. Conventional UA-SLE Method

An ultrasound-assisted solid–liquid extraction (UA-SLE) method, which was previously optimized for the analysis of the same Italian cultivar leaves [15], was also used in this study for comparison purposes. In this method, 0.100 g of the Italian mix sample was weighed in a beaker, and 10 mL of methanol:ultra-pure water (70:30, *v*/*v*) was added. The mixture was placed in the ultrasonic bath at room temperature for 15 min, and then the mixture was transferred to a centrifuge tube and centrifuged for 10 min at 2515× *g*. The supernatant was then placed in a separatory funnel, and 5 mL of hexane was added, in order to remove the chlorophylls contained in the leaves. Two phases were obtained: the top phase, containing the green pigments, and the hydroalcoholic phase in the bottom, containing the extracted phenolic compounds. The latter phase was collected and evaporated in the rotary evaporator, at 50 °C, until a volume of 1 mL was obtained. Finally, the solution was poured into a graduate cylinder, and methanol was added to reach a final volume of 2 mL. Before the injection in the HPLC-PDA system, the extract was filtered by using a PVDF syringe filter.

## 4. Conclusions

The proposed MA-SLE-HPLC-PDA method was successfully developed for the determination of phenolic compounds from *Vitis vinifera* leaves. The method was optimized by using an experimental design and only requires an aqueous solution of the C_16_C_4_Im-*Br* IL-based surfactant in a low concentration, due to its low CMC in the MA-SLE procedure, followed by filtration and direct injection of the extract in the HPLC system. The method resulted in being more effective, simpler, and eco-friendlier in comparison with a more conventional UA-SLE method, particularly due to the absence of organic solvent or cleanup steps in the whole procedure. As an additional feature, the aqueous extractant medium avoids co-extraction of chlorophylls and other non-polar interfering compounds from the leaves. Moreover, the method also presented greener features compared to other strategies reported in the literature dealing with the use of ILs or MA methods for the analysis of plant materials.

The proposed methodology was used to determine and compare the phenolic composition of Italian and Canarian grapevine leaves varieties. The results showed that the distribution of the phenolic markers follows a similar pattern in all the cultivars, with QUGlucur and QU being the most and least abundant compounds in the samples, respectively. In general, the Canarian varieties exhibited slightly higher total phenolic content, in comparison with the Italian cultivars and other varieties reported in the literature. Considering the previous studies on the potential of grapevine leaves as a source of antioxidant compounds, these results emphasize that these by-products are a source of nutraceutical compounds independently of the variety and geographic origin.

## Figures and Tables

**Table 1:** Phenolic content in the different *Vitis vinifera* varieties analyzed in this study, expressed as mg·g^−1^ ± SD (for three replicates) and using the MA-SLE method and the US-SLE method for comparison purposes, together with the amounts reported in the literature for other varieties and using different methods.

*Vitis vinifera* Variety (Method)	CA	RU	QUGlucos	QUGlucur	QU
**This Study**					
Piedmont, Italian (US-SLE-HPLC-PDA)	7.2 ± 0.9	0.6 ± 0.1	5 ±1	17 ± 2	0.27 ± 0.01
Piedmont, Italian (MA-SLE-HPLC-PDA)	9.8 ± 0.5	0.9 ± 0.1	10.3 ± 0.4	32.1 ± 0.4	0.19 ± 0.01
ML, Canarian (MA-SLE-HPLC-PDA)	7.7 ± 0.3	4.8 ± 0.2	8.1 ± 0.6	34 ± 2	0.25 ± 0.01
MA, Canarian (MA-SLE-HPLC-PDA)	11 ± 2	1.4 ± 0.1	10 ± 1	65 ± 9	0.20 ± 0.01
LB, Canarian (MA-SLE-HPLC-PDA)	9.7 ± 0.9	1.05 ± 0.07	7 ± 1	38± 3	0.26 ± 0.07
T, Canarian (MA-SLE-HPLC-PDA)	13.1 ± 0.7	3.3 ± 0.1	5.5 ± 0.2	62 ± 9	0.25 ± 0.02
NM, Canarian (MA-SLE-HPLC-PDA)	12.4 ± 0.7	1.9 ± 0.3	9 ± 1	39 ± 3	0.26 ± 0.01
LN, Canarian (MA-SLE-HPLC-PDA)	10.0 ± 0.5	1.57 ± 0.05	3.4 ± 0.4	21 ± 1	0.23 ± 0.01
**Values Reported in the Literature**					
Serbian (SLE-HPLC-MS/MS) ^a^	–	0.83 ± 0.02	5.8 ± 0.2	–	0.13 ± 0.03
Calabrian, Italian (US-SLE-HPLC-PDA) ^b^	–	0.10 ± 0.01	–	–	0.15 ± 0.01
Brazilian (SLE-HPLC-MS/MS) ^c^	–	0.03	0.82	3.78	–

^a^ Mean values for nine different Serbian cultivars [39]. ^b^ Mean values for six different Calabrian cultivars [38]. ^c^ Mean values for four different Brazilian cultivars [37]. Listán Negro, LN; Negra Moll, NM; Tintilla, T; Listán Blanco, LB; Malvasía Lanzarote, ML; Moscatel Alejandría, MA.

## Supplementary Materials

The following are available online. Figure S1: Chemical structure and main characteristics of the IL-based surfactants used in this study. Figure S2: Representative chromatograms obtained for the analysis of the Piedmont leaves (Italian cultivar mix), using the proposed MA-SLE-HPLC-PDA method with the C16C4Im-*Br* IL-based surfactant, and the UA-SLE-HPLC-PDA method (reproduced from Acquadro et al., 2020). There is an offset of 4% in the signal axis to overlap the chromatograms. (1) CA, (2) RU, (3) QUGlucos, (4) QUGlucur, and (5) QU. Figure S3: Phenolic composition of the different *Vitis vinifera* varieties analyzed in this study with the proposed MA-SLE method, using the C16C4Im*-Br* IL-based surfactant. Figure S4: Results obtained with different statistical analysis, using the data obtained for the analysis of Canarian cultivars with the proposed method: (A) Scatterplot of the scores obtained for the different varieties of Canarian cultivars by Principal Component Analysis (PCA). (B) Loading plot showing the weights of the phenolic compound on the principal components. (C) Correlation between the Canarian varieties and the phenolic content by hierarchical clustering (HCA), using one minus Pearson’s correlation coefficient. Table S1: Design matrix for the screening analysis, a 2^3^ factorial design and three central points, in the optimization of the MA-SLE method. Table S2: Matrix of the experiments of the Doehlert design used in the optimization of the MA-SLE method, including the coded values and the operating values. Table S3: Several quality analytical parameters of the HPLC-PDA method. Table S4: *Vitis vinifera* leaves varieties from Canary Islands, employed for the determination and quantification of phenolic markers. Table S5: Chemical structures and physicochemical properties of the phenolic compounds determined in this study, obtained from SciFinder^®^ 2020 database.
