# Supplementary material for: Sustainable Micro-Scale Extraction of Bioactive Phenolic Compounds from Vitis vinifera Leaves with Ionic Liquid-Based Surfactants
Source: Molecules. 2020 Jul 6;25(13):3072. doi: 10.3390/molecules25133072 (PMC7412462; doi:10.3390/molecules25133072)
Supplement: Supplementary file 1 [file molecules-25-03072-s001.pdf]

SUPPLEMENTARY MATERIAL

Sustainable micro-scale extraction of bioactive phenolic compounds from *Vitis vinifera* leaves with ionic liquid-based surfactants

Giulia Mastellone[1,2], Idaira Pacheco-Fernández[2,3], Patrizia Rubiolo[1], Verónica Pino[2,3], Cecilia Cagliero[1*]

[1] Dipartimento di Scienza e Tecnologia del Farmaco, Università degli Studi di Torino, I-10125, Torino, Italy; giulia.mastellone@edu.unito.it (G.M.), patrizia.rubiolo@unito.it (P.R.), cecilia.cagliero@unito.it (C.C.)
[2] Laboratorio de Materiales para Análisis Químicos (MAT4LL), Departamento de Química, Unidad Departamental de Química Analítica, Universidad de La Laguna (ULL), Tenerife, 38206, Spain; ipacheco@ull.edu.es (I.P.-F.), veropino@ull.edu.es (V.P.)
[3] Instituto Universitario de Enfermedades Tropicales y Salud Pública de Canarias, Universidad de La Laguna (ULL), Tenerife, 38206, Spain
** Correspondence: cecilia.cagliero@unito.it (C.C.)*

Table of Contents

Figures

Figure S1 .... page S2
Figure S2 .... page S3
Figure S3 .... page S4
Figure S4 .... page S5

Tables

Table S1 .... page S6
Table S2 .... page S7
Table S3 .... page S8
Table S4 .... page S9
Table S5 .... page S10

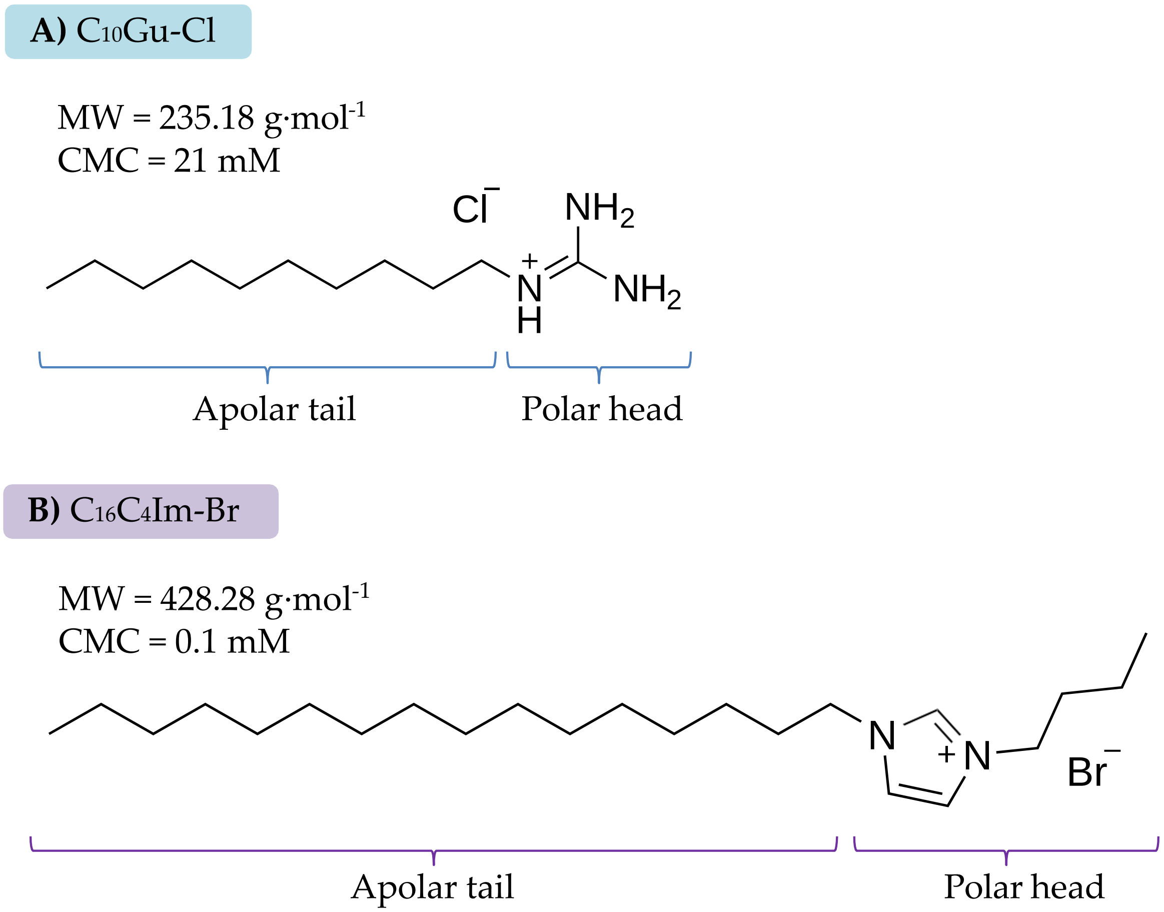

Figure S1. Chemical structure and main characteristics of the IL-based surfactants used in this study.

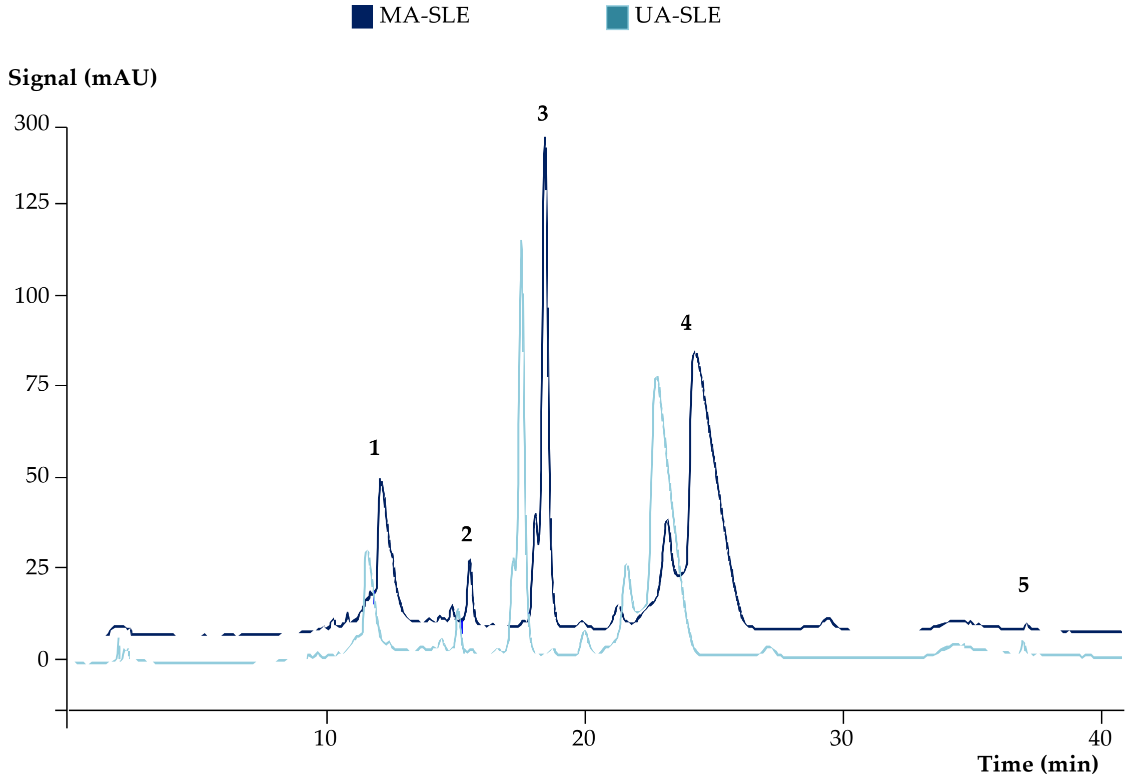

Figure S2. Representative chromatograms obtained for the analysis of the Piedmont leaves (Italian cultivar mix) using the proposed MA-SLE-HPLC-PDA method with the $C_{16}C_4Im$- Br IL-based surfactant, and the UA-SLE-HPLC-PDA method (reproduced from Acquadro *et al.*, 2020). There is an offset of 4% in the signal axis to overlap the chromatograms. 1: CA, 2: RU, 3: QUGlucos, 4: QUGlucur, 5: QU.

Acquadro, S., Appleton, S., Marengo, A., Bicchi, C., Sgorbini, B., Mandrone, M., Gai, F., Peiretti, P. G., Cagliero, C., & Rubiolo, P. (2020). Grapevine Green Pruning Residues as a Promising and Sustainable Source of Bioactive Phenolic Compounds. *Molecules*, *25*, 464.

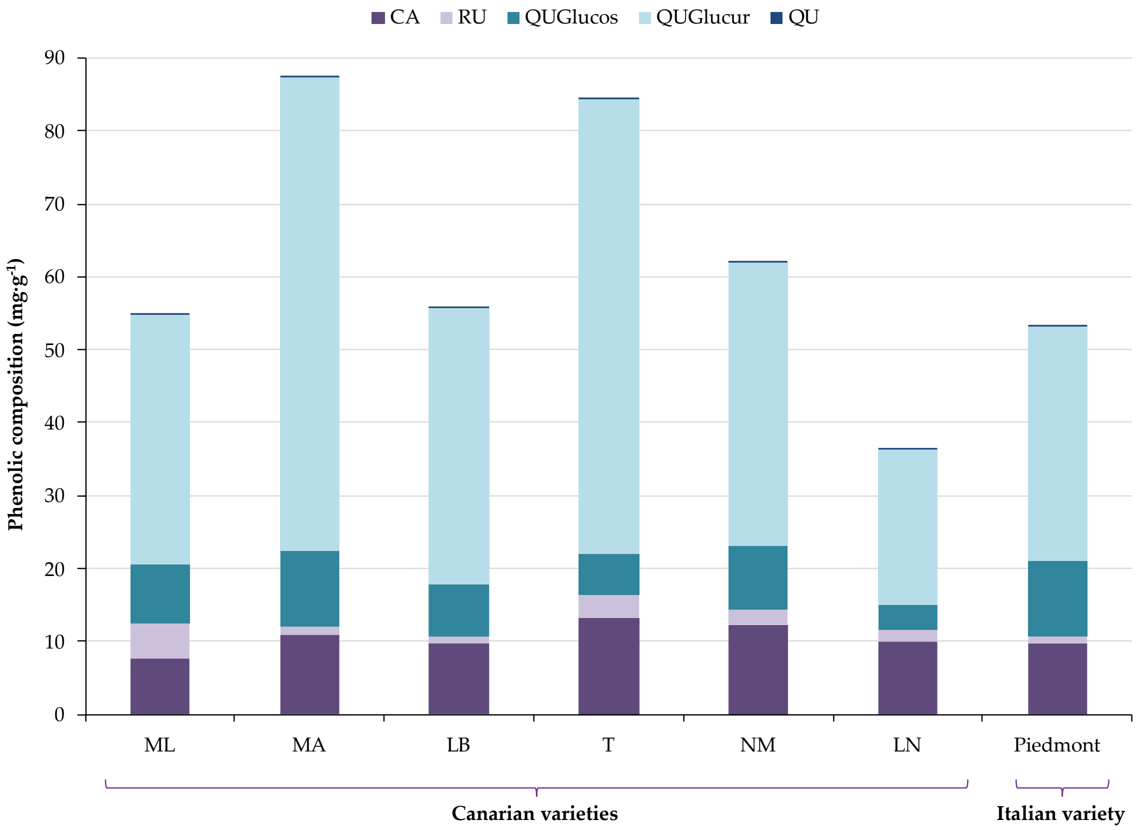

Figure S3. Phenolic composition of the different *Vitis vinifera* varieties analyzed in this study with the proposed MA-SLE method using the $C_{16}C_4$Im-Br IL-based surfactant.

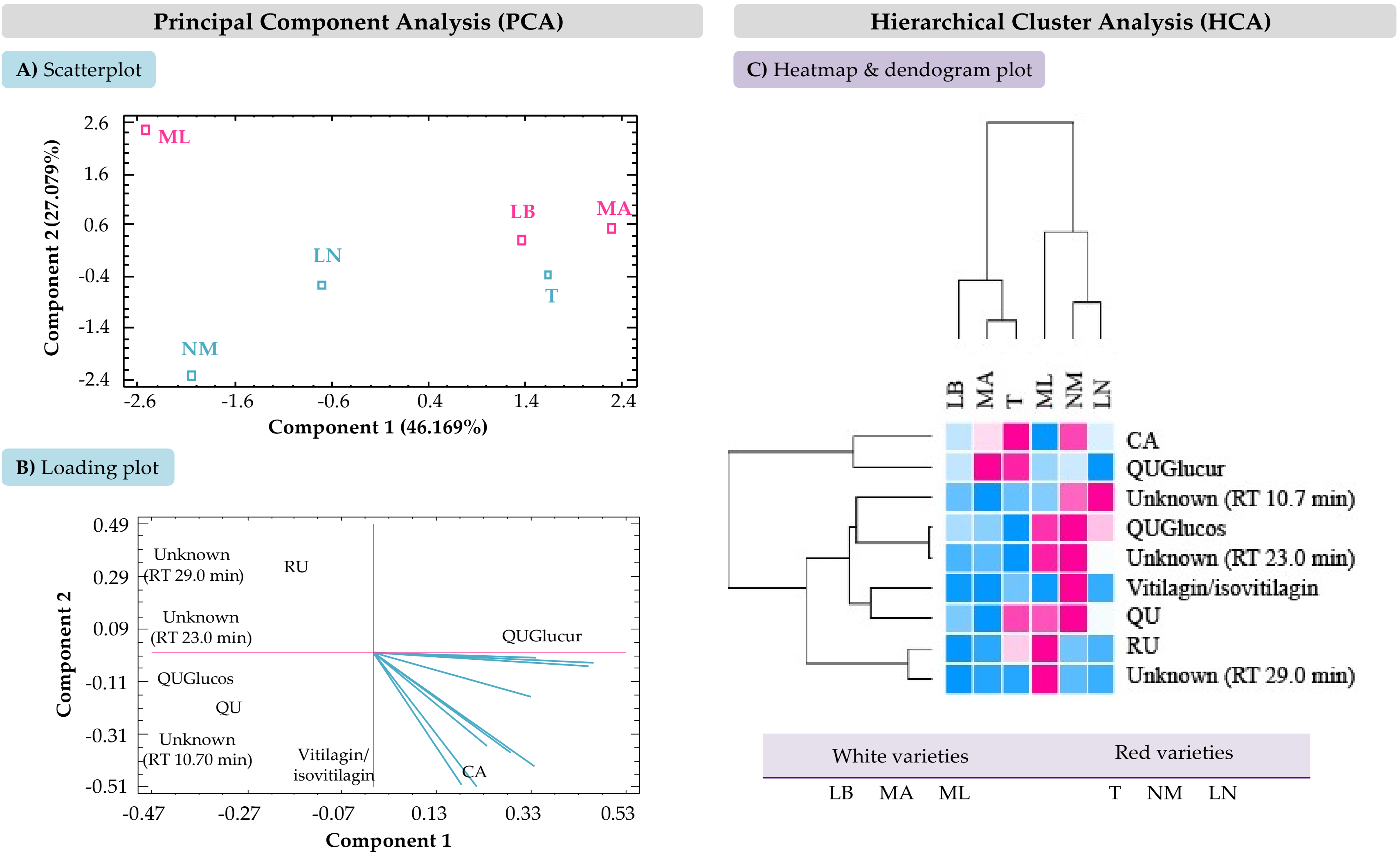

Figure S4. Results obtained with different statistical analysis using the data obtained for the analysis of Canarian cultivars with the proposed method: **A)** Scatterplot of the scores obtained for the different varieties of Canarian cultivars by Principal Component Analysis (PCA). **B)** Loading plot showing the weights of the phenolic compound on the principal components. **C)** Correlation between the Canarian varieties and the phenolic content by hierarchical clustering (HCA) using one minus Pearson's correlation coefficient.

Table S1. Design matrix for the screening analysis, a 2^3 factorial design and 3 central points, in the optimization of the MA-SLE method.

Experiment	IL-based surfactant concentration (mM)	MW temperature (℃)	MW time (min)
1	2.5	55	15
2	2.5	55	40
3	2.5	75	15
4	2.5	75	40
5	12.5	55	15
6	12.5	55	40
7	12.5	75	15
8	12.5	75	40
9	7.5	65	27.5
10	7.5	65	27.5
11	7.5	65	27.5

Table S2. Matrix of the experiments of the Doehlert design used in the optimization of the MA-SLE method, including the coded values and the operating values.

Experiment	IL-based surfactant concentration (mM)		MW time (min)	
	**C_1**	**X_1**	**C_2**	**X_2**
1	0	1.3	0	32.5
2	1	2.5	0	32.5
3	0.5	1.9	0.866	50
4	-1	0.1	0	32.5
5	-0.5	0.7	-0.866	15
6	0.5	1.9	-0.866	15
7	-0.5	0.7	0.866	50
8	0	1.3	0	32.5
9	0	1.3	0	32.5

C_1 and C_2 are the coded values for the levels of IL-based surfactant concentration (mM) and time of MW treatment (min), respectively.

The relationship between coded and real values is given by: $C_i = \left(\frac{X_i - X_i^o}{\Delta X_i}\right) \alpha$

where C_i is the coded value for the level of factor i, X_i is its real value in an experiment, X^o_i is the real value at the center of the experimental domain, ΔX_i is the step of variation of the real value, and α is the coded value limit for each factor.

The number of experiments required (N) is given by $N = k^2 + k + C_0$, where k is the number of variables and C_0 is the number of center points.

Table S3. Several quality analytical parameters of the HPLC-PDA method.

Compound	λ^a (nm)	Linear range ($mg\cdot L^{-1}$)	Slope ± SD[b]	$S_{y/x}$[c]	R^2 [d]	LOD[e] ($mg\cdot L^{-1}$)	LOQ[f] ($mg\cdot L^{-1}$)
CA	320	5 – 650	11154 ± 664	272	0.997	3.0	5.0
RU	360	5 – 500	19604 ± 2422	872	0.992	1.0	3.3
QUGlucos	360	5 – 500	13460 ± 513	199	0.999	1.0	3.3
QUGlucur	360	5 – 500	5560 ± 283	110	0.998	1.0	3.3
QU	360	5 – 500	20014 ± 937	338	0.999	0.5	1.7

[a] wavelength used for quantification

[b] standard deviation within the calibration range for n = 7 calibration levels

[c] standard deviation of the residuals or error of the estimate

[d] determination coefficient

[e] limit of detection, determined by decreasing the concentration of the standards until a S/N ratio of 3 was obtained

[f] limit of quantification, estimated as 10/3 times the LOD, and experimentally verified using standards at the predicted concentrations

Tables S4. *Vitis vinifera* leaves varieties from Canary Islands, employed for the determination and quantification of phenolic markers.

**Variety name (abbreviation)**	**Classification**	**Leaf anatomy**[a]
Malvasía Lanzarote (ML)	White v.	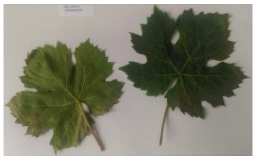
Moscatel Alejandría (MA)	White v.	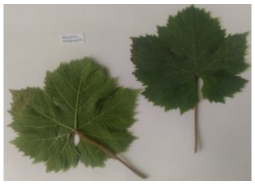
Listán Blanco (LB)	White v.	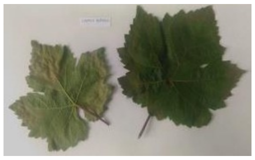
Tintilla (T)	Red v.	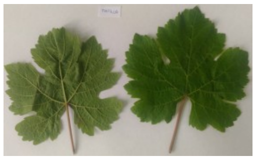
Negro Moll (NM)	Red v.	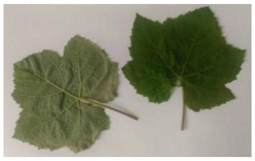
Listán Negro (LN)	Red v.	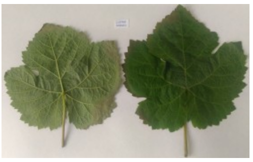

[a] photos of the samples used in the study

Table S5. Chemical structures and physicochemical properties of the phenolic compounds determined in this study, obtained from SciFinder® 2020 database.

Analyte	Chemical structure	Type	MW (g·mol^{-1})	pK_a	Log K_{ow}[a]
CA	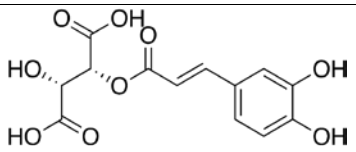	phenolic acid	312.23	2.18	1.15
RU	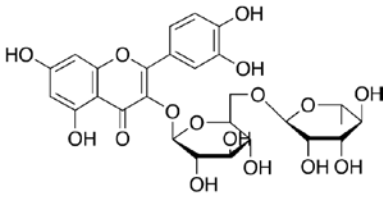	flavonoid	610.52	6.17	-0.90
QUGlucos	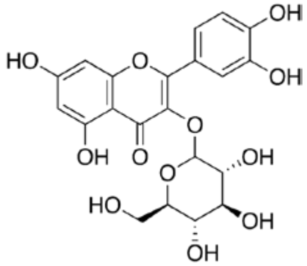	flavonoid	464.38	6.17	-0.11
QUGlucur	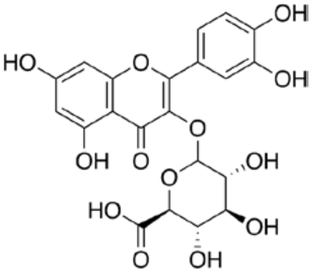	flavonoid	478.36	2.76	0.62
QU	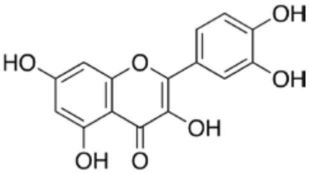	flavonoid	302.24	6.31	1.99

[a] logarithm of octanol/water partition coefficient at 25 ºC.
